# UDP-Glucuronic Acid Transport Is Required for Virulence of *Cryptococcus neoformans*

**DOI:** 10.1128/mBio.02319-17

**Published:** 2018-01-30

**Authors:** Lucy X. Li, Carsten Rautengarten, Joshua L. Heazlewood, Tamara L. Doering

**Affiliations:** a Department of Molecular Microbiology, Washington University School of Medicine, St. Louis, Missouri, USA; b School of Biosciences, University of Melbourne, Melbourne, Australia; Duke University Medical Center

**Keywords:** *Cryptococcus neoformans*, UDP-glucuronic acid, fungal pathogenesis, glycan synthesis, nucleotide sugar transporter, polysaccharide capsule

## Abstract

Glycans play diverse biological roles, ranging from structural and regulatory functions to mediating cellular interactions. For pathogens, they are also often required for virulence and survival in the host. In *Cryptococcus neoformans*, an opportunistic pathogen of humans, the acidic monosaccharide glucuronic acid (GlcA) is a critical component of multiple essential glycoconjugates. One of these glycoconjugates is the polysaccharide capsule, a major virulence factor that enables this yeast to modulate the host immune response and resist antimicrobial defenses. This allows cryptococci to colonize the lung and brain, leading to hundreds of thousands of deaths each year worldwide. Synthesis of most glycans, including capsule polysaccharides, occurs in the secretory pathway. However, the activated precursors for this process, nucleotide sugars, are made primarily in the cytosol. This topological problem is resolved by the action of nucleotide sugar transporters (NSTs). We discovered that Uut1 is the sole UDP-GlcA transporter in *C. neoformans* and is unique among NSTs for its narrow substrate range and high affinity for UDP-GlcA. Mutant cells with *UUT1* deleted lack capsule polysaccharides and are highly sensitive to environmental stress. As a result, the deletion mutant is internalized and cleared by phagocytes more readily than wild-type cells are and is completely avirulent in mice. These findings expand our understanding of the requirements for capsule synthesis and cryptococcal virulence and elucidate a critical protein family.

## IMPORTANCE

*Cryptococcus neoformans* causes lethal meningitis in almost two hundred thousand immunocompromised patients each year. Much of this fungal pathogen’s ability to resist host defenses and cause disease is mediated by carbohydrate structures, including a complex polysaccharide capsule around the cell. Like most eukaryotic glycoconjugates, capsule polysaccharides are made within the secretory pathway, although their precursors are generated in the cytosol. Specific transporters are therefore required to convey these raw materials to the site of synthesis. One precursor of particular interest is UDP-glucuronic acid, which donates glucuronic acid to growing capsule polysaccharides. We discovered a highly specific, high-affinity transporter for this molecule. Deletion of the gene encoding this unusual protein abolishes capsule synthesis, alters stress resistance, and eliminates fungal virulence. In this work, we have identified a novel transporter, elucidated capsule synthesis and thereby aspects of fungal pathogenesis, and opened directions for potential antifungal therapy.

## INTRODUCTION

UDP-glucuronic acid (UDP-GlcA) is a critical precursor for essential glycoconjugates across biological kingdoms, ranging from mammalian glycosaminoglycans and plant cell wall polysaccharides to bacterial capsule glycoglycerolipids (1–3). Aberrant UDP-GlcA synthesis or subcellular localization leads to severe impairments such as Schneckenbecken dysplasia in humans (4) and virulence defects in bacterial pathogens (5–7).

Our interest in UDP-GlcA stems from its role in the fungal pathogen *Cryptococcus neoformans*. This opportunistic yeast colonizes the lungs and disseminates to the brains of immunocompromised individuals, where it causes meningoencephalitis that is responsible for roughly two hundred thousand deaths per year (8–10). UDP-GlcA is a key biosynthetic precursor of cryptococcal polysaccharides. These complex polymers associate with the cell wall to form the cryptococcal capsule, which provides a physical barrier against host immune defenses. These polysaccharides are also shed into the extracellular space (11), where they impede host defenses by interfering with phagocytosis and clearance of the yeast, inhibiting the production of proinflammatory cytokines, depleting complement components, and reducing leukocyte migration to sites of inflammation (12).

The capsule consists of two complex polysaccharides, glucuronoxylomannan (GXM) and glucuronoxylomannogalactan (GXMGal). GXM, which constitutes 90% of the capsule by mass, is a repeating polymer with a mannose (Man) backbone that is partially acetylated and is substituted with monosaccharide side chains of glucuronic acid (GlcA) and xylose (Xyl) (13). The remaining 10% of the capsule mass is made up of GXMGal, which is a linear galactose (Gal) polymer bearing both single galactofuranose (Gal*f*) residues and galactomannan side chains substituted with a variable number of GlcA and Xyl residues (14–16). Overall, GlcA comprises roughly 16% and 7% of the residues in GXM and GXMGal, respectively, and is responsible for their acidic nature.

Both GXM and GXMGal, like most other eukaryotic glycans, are believed to be assembled in the secretory pathway (17). However, nucleotide sugars, which donate individual sugar moieties to growing glycan structures, are synthesized primarily in the cytosol (18). The endoplasmic reticulum (ER) and Golgi membranes, therefore, constitute physical barriers that prevent substrate access to biosynthetic enzymes. Nucleotide sugar transporters (NSTs) provide a solution to this topological problem by translocating activated sugars into the luminal space in exchange for the corresponding nucleoside monophosphates (19, 20). In this way, NSTs enable luminal glycan biosynthesis.

Predicted protein sequence is not a reliable predictor of NST substrate specificity. For example, NSTs with almost 50% amino acid identity have been reported to transport distinct substrates, while others with only 20% identity appear to translocate the same substrate (21). Further complicating the picture, individual NSTs range from highly specific proteins that recognize a single substrate to less restrictive ones that transport up to four substrates, and NSTs with overlapping but nonidentical substrate affinities can be found in a single cell (22–28). Finally, associated glycan synthetic enzymes and subcellular localization may also influence the activity of a given NST (29, 30). *A*ll of these factors make it a challenge to identify and characterize the full complement of NSTs in a cell type of interest, impeding our ability to fully define critical glycan synthetic processes.

Mutant *C. neoformans* strains incapable of synthesizing UDP-GlcA do not produce capsule or cause disease in mice, demonstrating the importance of GlcA in cryptococcal biology and pathogenesis (31, 32). Despite this, the NST(s) responsible for transporting its donor, UDP-GlcA, has never been identified in *C. neoformans*. Here we show that *C. neoformans* Uut1 is a UDP-GlcA transporter by using an *in vitro* assay to directly demonstrate its activity; we also characterize its specificity and kinetic properties. We further show that cells lacking Uut1 exhibit marked growth defects and metabolic abnormalities, which correlate with greater phagocytosis by host macrophages and quicker clearance of infection *in vitro* and *in vivo*. Uut1 is thus a critical protein for cryptococcal biosynthetic processes and is required for multiple aspects of *C. neoformans* virulence.

## RESULTS

**FIG 1:**
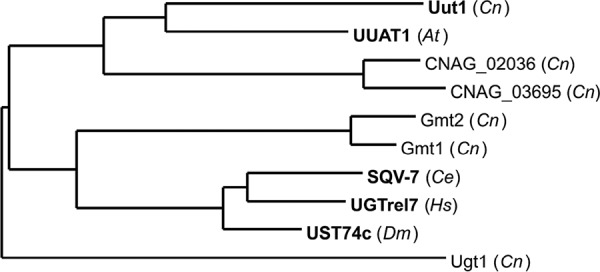
Evolutionary conservation of UDP-GlcA transporters. Phylogenetic relationships of *C. neoformans* (*Cn*) nucleotide sugar transporters (NSTs) and other UDP-GlcA transporters (shown in boldface type) from *Caenorhabditis elegans* (*Ce*), *Homo sapiens* (*Hs*), *Drosophila melanogaster* (*Dm*), and *Arabidopsis thaliana* (*At*). Tree reconstruction was performed with the Phylogeny.fr web server (62, 63) using MUSCLE, PhyML, and TreeDyn software. Branch lengths are drawn to scale.

To identify the cryptococcal UDP-GlcA transporter, we first used BLASTP to search the *C. neoformans* genome for predicted proteins with homology to known UDP-GlcA transporters. Although we found no homologs of the transporters from *Caenorhabditis elegans*, *Homo sapiens*, or *Drosophila melanogaster*, we did find a predicted ortholog of the *Arabidopsis thaliana* transporter UUAT1 (33), which we termed Uut1 (CNAG_06230). Similar to other NSTs, Uut1 is predicted to have an even number of transmembrane domains (here 10) such that the N and C termini are on the same side of the membrane, likely in the cytosol (see Fig. S1 in the supplemental material). Phylogenetic analysis of Uut1 places it closest to UUAT1 (Fig. 1), and more distant from other UDP-GlcA transporters and from known cryptococcal NSTs. Notably, Uut1 and UUAT1 share only 16% amino acid identity, although as discussed above, homology is a poor predictor of substrate specificity in this family of proteins.

10.1128/mBio.02319-17.1 FIG S1  Predicted secondary structure of Uut1 (550 amino acids), showing an extended N-terminal domain (amino acids 1 to 244), 10 predicted transmembrane domains, and a predicted ER localization signal (KXKXX motif; dark gray) near the C terminus. Download FIG S1, PDF file, 0.3 MB. Copyright © 2018 Li et al. 2018 Li et al. This content is distributed under the terms of the Creative Commons Attribution 4.0 International license.

**FIG 2:**
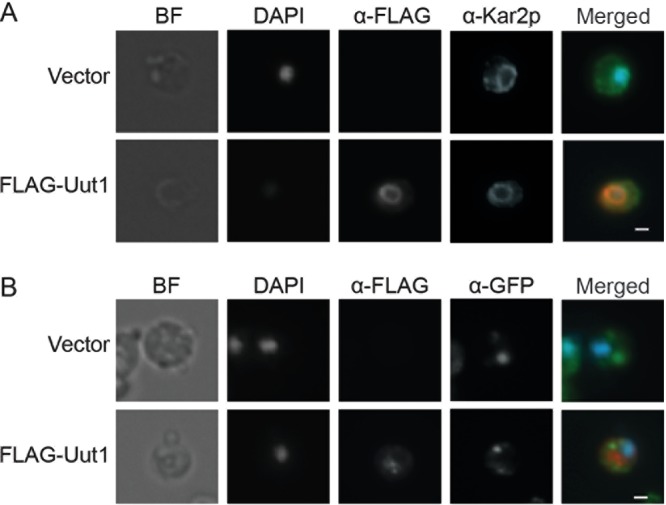
Cryptococcal Uut1 colocalizes with the ER marker Kar2p/BiP (A), but not with the late Golgi marker Sec7 (B). *S. cerevisiae* cells expressing Sec7-3xGFP (GFP stands for green fluorescent protein) transformed with vector alone (Vector) or vector expressing FLAG-tagged Uut1 (FLAG-Uut1) were stained with DAPI and probed with the indicated antibodies. Bright-field (BF), single-channel, and merged images are shown, and all images have the same magnification (bars, 1 μm). The colors indicate the following: blue, DAPI; red, anti-FLAG (α-FLAG); green, anti-GFP (α-GFP) and anti-Kar2p/BiP (α-Kar2p). Images are representative of three independent immunofluorescence experiments.

If Uut1 is indeed a nucleotide sugar transporter that supplies precursors for polysaccharide synthesis, we expect it to reside in the secretory pathway. To test this, we took advantage of existing markers and facile imaging methods in the model yeast *Saccharomyces cerevisiae* and expressed FLAG-tagged Uut1 (FLAG-Uut1) under a copper-inducible promoter in that system. Immunofluorescence (IF) staining showed that the tagged protein appeared to colocalize with an ER marker (Kar2p/BiP) but not with the late Golgi marker Sec7 (34) (Fig. 2; see Discussion). A KXKXX motif near the C terminus (Fig. S1) may be involved in this localization. Such motifs mediate the retrieval of type I transmembrane proteins from downstream membranes to the ER (35, 36), although the Uut1 sequence (Fig. S1) is atypical in that it is followed by three additional amino acids.

**FIG 3:**
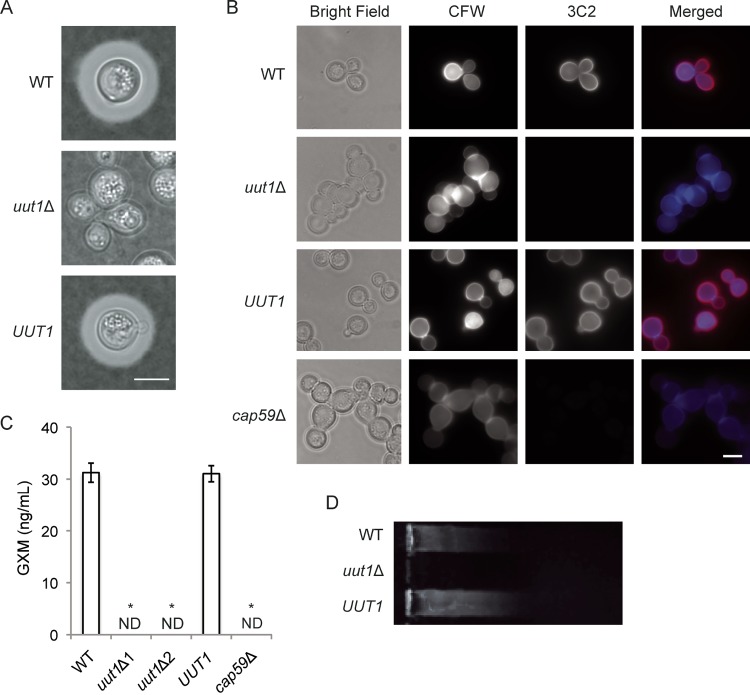
The *uut1*Δ mutant does not produce capsule. (A) Wild-type (WT), *uut1*Δ, and complemented *uut1*Δ (*UUT1*) strains were cultured under capsule-inducing conditions (see Materials and Methods) for 24 h and then visualized by light microscopy after negative staining with India ink. Bar = 5 μm. (B) Cells from the indicated strains were incubated with calcofluor white (CFW; blue) to stain the cell wall and with MAb 3C2 (red) to visualize the capsule. Bright-field, single-channel gray scale, and merged images are shown. Bar = 5 μm. (C) Shed capsule polysaccharide, from two independent deletions and control strains, was quantitated by ELISA (see Materials and Methods). Values are means ± standard errors of the means (SEMs) (error bars) from three independent experiments. Values that are significantly different (*P* < 0.01) by one-way ANOVA and Tukey’s posthoc test from the value for the WT strain are indicated by an asterisk. ND, not detected. (D) Conditioned medium from the indicated strains was resolved on an agarose gel, transferred to a nylon membrane, and analyzed by immunoblotting with anti-GXM MAb 3C2 as in reference 69.

To define the role of Uut1 in cryptococcal biology, we deleted the corresponding gene (*UUT1*). Even when the resulting mutant was grown in capsule-inducing conditions (see Materials and Methods), we detected no capsule by negative staining (Fig. 3A) or by staining with fluorophore-conjugated anti-GXM antibodies (Fig. 3B). We also detected no GXM shed into the growth medium by enzyme-linked immunosorbent assay (ELISA) (Fig. 3C) or by immunoblotting (Fig. 3D), and the mutant cells appeared clumpy compared to the wild type (WT) (Fig. 3A and B). All of these phenotypes were consistent with those of the acapsular *cap59*Δ strain and were reversed when the deletion was complemented with the wild-type gene at the original locus (Fig. 3, *UUT1*). We obtained similar results for capsule staining, GXM shedding ELISA, and GXM immunoblotting with additional antibodies (Table S1). As far as we can determine, therefore, cells lacking Uut1 are completely acapsular.

10.1128/mBio.02319-17.8 TABLE S1  Summary of GXM detection assays. Download TABLE S1, PDF file, 0.04 MB. Copyright © 2018 Li et al. 2018 Li et al. This content is distributed under the terms of the Creative Commons Attribution 4.0 International license.

The absence of capsule on cells lacking Uut1 suggested that this putative nucleotide transporter translocates a major capsule substrate(s). The components of GXM and GXMGal are Man, Gal, Xyl, Gal*f*, and GlcA. We previously identified two transporters of GDP-Man (30, 37), and there is a known UDP-Gal transporter (38, 39), so we hypothesized that those were less likely to be substrates of Uut1. Additionally, even completely abrogating synthesis of UDP-Xyl or UDP-Gal*f* yields hypo- or normocapsular cells, respectively (15, 32, 40), rather than the acapsular cells observed for the *uut1*Δ mutant; this argued against these capsule donors as Uut1 substrates. This reasoning left UDP-GlcA as the best candidate substrate, which was further supported by the observation that cells unable to synthesize UDP-GlcA are acapsular (31, 32).

**FIG 4:**
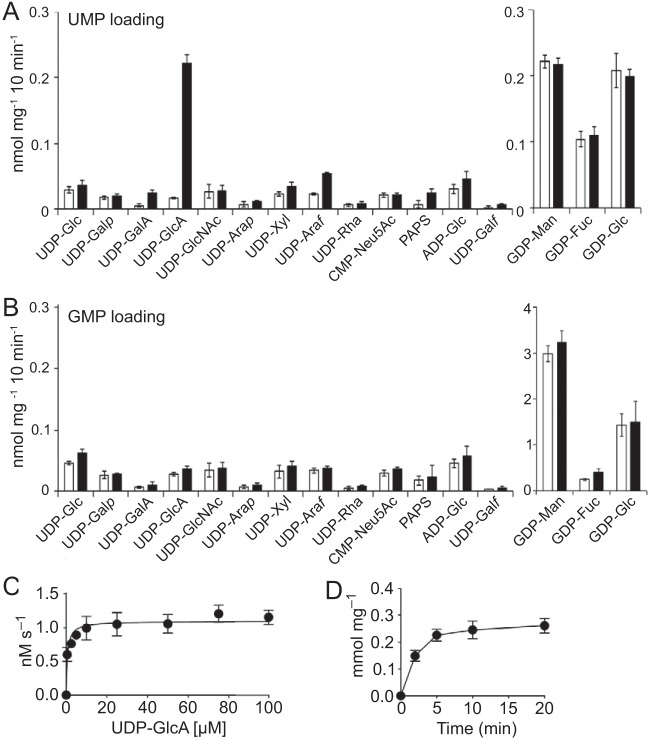
Uut1 activity *in vitro*. (A and B) Substrate and exchange substrate specificity of Uut1. Proteoliposomes from cells without Uut1 (white bars) or with Uut1 (black bars), preloaded with 30 mM UMP (A) or 30 mM GMP (B), were incubated for 10 min with a mixture of the indicated nucleotide sugars, each at 50 μM. Data were normalized to the total protein content of the proteoliposome preparations. (C) Proteoliposomes from cells expressing Uut1, preloaded with 10 mM UMP, were incubated with UDP-GlcA at the indicated concentrations for 2 min, and UDP-GlcA transport was measured as described in Materials and Methods. (D) Proteoliposomes, preloaded as described above for panel C, were assayed with 50 μM UDP-GlcA for the times shown. Values are normalized to the Uut1 content of the proteoliposome preparations (see Table S2 in the supplemental material). All values shown are the means ± SEMs (error bars) from four independent experiments.

To test our hypothesis that Uut1 transports UDP-GlcA, we directly assayed its activity *in vitro*. For these studies, we first prepared microsomes from *S. cerevisiae* heterologously expressing V5-tagged Uut1. We then reconstituted the microsomal protein in proteoliposomes, which were preloaded with UMP or GMP to serve as antiport substrates. After confirmation of Uut1-V5 expression by immunoblotting (Fig. S2), the proteoliposomes were incubated with a mixture of nucleotide sugars, subjected to gel filtration to remove any that were not imported, and analyzed by liquid chromatography-tandem mass spectrometry (LC-MS/MS). UDP-GlcA was the only cryptococcal nucleotide sugar that was transported over background by Uut1-bearing proteoliposomes preloaded with UMP (Fig. 4A). This transport was saturable with time and substrate concentration (Fig. 4C and D) and had an apparent *K*_*m*_ of 0.6 ± 0.1 μM and *V*_max_ of 1.1 ± 0 nM s^−1^ (mean ± standard error of the mean [SEM] from four independent experiments) with a turnover rate of 0.08 s^−1^. We also observed minor transport of UDP-galacturonic acid and UDP-arabinofuranose, but those substrates have never been reported in *C. neoformans* and were not detected in our analyses (see below). We observed no transport of any assayed nucleotide sugar when the proteoliposomes were preloaded with GMP (Fig. 4B).

10.1128/mBio.02319-17.2 FIG S2  Anti-V5 immunoblot of proteoliposomes prepared from *S. cerevisiae* expressing vector alone (control) or V5-tagged Uut1; 2.5 μg of total protein was loaded into each lane. The positions of molecular mass standards (in kilodaltons) are shown to the left of the gel. In *S. cerevisiae*, the expressed and active polypeptide is cleaved prior to amino acid 200. Download FIG S2, PDF file, 0.2 MB. Copyright © 2018 Li et al. 2018 Li et al. This content is distributed under the terms of the Creative Commons Attribution 4.0 International license.

**FIG 5:**
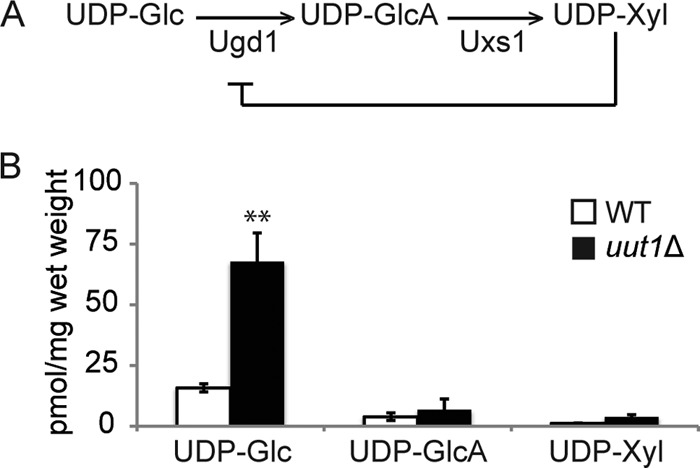
UDP-GlcA metabolism. (A) UDP-GlcA synthesis and regulation. (B) Nucleotide sugar concentrations in the *uut1*Δ mutant (black bars) compared to the WT control (white bars). Values are the averages ± SEMs for four independent replicates. Values that are significantly different (*P* ≤ 0.01) by two-tailed Student’s *t* test are indicated by two asterisks.

We next wondered whether eliminating UDP-GlcA transport would alter cellular nucleotide sugar metabolism. UDP-GlcA is synthesized from UDP-Glc by UDP-Glc dehydrogenase (Ugd1), and it may be decarboxylated by UDP-Xyl synthase (Uxs1) to produce UDP-Xyl; this product inhibits Ugd1 to regulate the pathway (Fig. 5A). Our measurements of nucleotide sugar content (see Materials and Methods) showed that the level of UDP-Glc in *uut1*Δ cells was about 4-fold higher than in WT cells (Fig. 5B). The levels of UDP-GlcA and UDP-Xyl, in contrast, were not significantly different in the mutant and WT cells (Fig. 5B).

**FIG 6:**
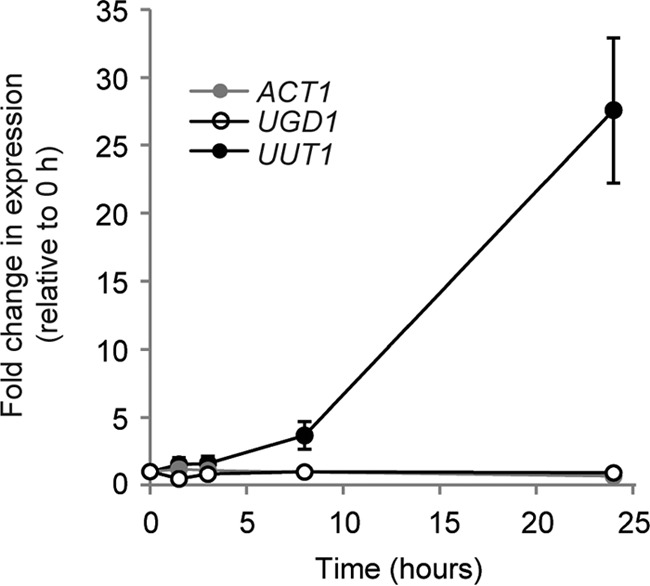
Transcription of *UUT1* increases during capsule induction. The numbers of reads from RNA-seq data (mean ± SD) during capsule induction (see Materials and Methods) were normalized to their levels at *t* = 0, which were as follows: 125,691 ± 8,645 for *UUT1*, 1,761,976 ± 108,920 for *UGD1*, and 4,495,647 ± 279,579 for *ACT1* (included as a control). Values shown are compiled from three independent experiments, each with RNA prepared from three biological replicates as in reference 74.

We next examined the cellular level of UDP-GlcA when *C. neoformans* was incubated for 24 h under conditions that induce capsule synthesis, which we expected to require increased UDP-GlcA. Surprisingly, the overall concentration of UDP-GlcA remained constant under these conditions (4 ± 2 pmol/mg [wet weight]; mean ± standard deviation [SD] from four independent experiments). Consistent with this observation, transcriptome sequencing (RNA-seq) studies showed no change in *UGD1* transcription over this interval (Fig. 6). However, under the same conditions, *UUT1* expression was upregulated 28-fold (Fig. 6). It thus appears that the increased demand for UDP-GlcA is satisfied by greater transport in the context of adequate cytosolic pools.

GXM and GXMGal are the only *C. neoformans* glycans known to contain glucuronic acid, although not all cryptococcal glycoconjugates have been extensively profiled. Furthermore, the UDP-GlcA synthase mutant, the *ugd1*Δ mutant, exhibits profound cellular defects that cannot be solely attributed to the absence of capsule (31, 32). We therefore assayed additional characteristics of cells lacking Uut1.

**FIG 7:**
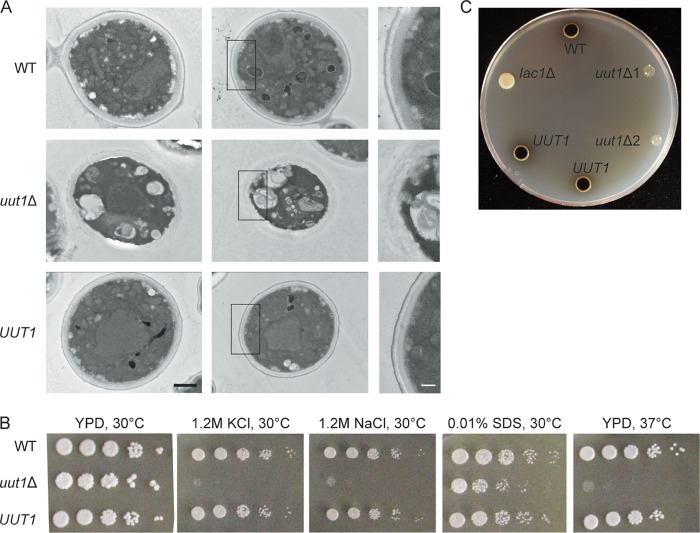
*uut1*Δ mutants exhibit defects in cell morphology and growth. (A) Electron micrographs of WT, *uut1*Δ, and *UUT1* strains grown in rich medium (left and middle columns, bar = 1 μm) with enlarged insets (right column, bar = 0.25 μm). (B) Melanization of the indicated strains after growth on l-DOPA plates (see Materials and Methods). *lac1*Δ cells do not melanize (75). (C) The indicated strains were grown overnight at 30°C in YPD medium, and 5-μl volumes of serially diluted strains (10-fold serial dilutions starting at 10^6^ cells/ml) were spotted and grown on YPD or minimal medium under the conditions shown. Images of the 30°C and 37°C plates were taken 2 and 3 days after inoculation, respectively.

We had already noticed that *ugd1*Δ mutant cells appeared smaller and less spherical than WT cells and exhibited the aggregation typical of acapsular strains (Fig. 3A). Closer examination of the *uut1*Δ mutant by transmission electron microscopy (TEM) confirmed these observations, and revealed the absence of the distinct morphological layers in the cell wall (Fig. 7A) that are normally present in WT cells (11, 41–43). The mutant cell wall also appeared less organized (Fig. 7A) and showed altered exposure of mannans as detected by ConA binding, although dyes recognizing other components of the cell wall bound the two strains similarly (Fig. S3). Furthermore, the cell membrane appeared to make irregular contact with the internal surface of the cell wall, and the cells contained large vacuoles and abnormal intracellular inclusions, often associated with the plasma membrane, whether they were grown in rich or nutrient-deficient media (Fig. 7A and Fig. S4). The contents of vacuoles or membranous inclusions were not recognized by anti-GXM antibody in immunoelectron microscopy studies (Fig. S4).

10.1128/mBio.02319-17.3 FIG S3  Surface exposure of cell wall components. WT, *uut1*Δ, *UUT1*, and *cap59*Δ strains were grown and stained with CFW (binds chitin), concanavalin A (binds mannoproteins), eosin Y (binds chitosan), and Pontamine (binds unspecified cell wall components). The images shown are representative of two independent experiments. Bar = 5 μm. Download FIG S3, PDF file, 0.8 MB. Copyright © 2018 Li et al. 2018 Li et al. This content is distributed under the terms of the Creative Commons Attribution 4.0 International license.

10.1128/mBio.02319-17.4 FIG S4  GXM is not detected within or around *uut1*Δ cells, in contrast to the abundant labeling of this capsule component on WT and complemented controls. Shown are electron micrographs of WT, *uut1*Δ, and *UUT1* strains grown for 24 h in nutrient-deficient media, which induces capsule production. Sections were labeled with anti-GXM MAb 3C2 and 12-nm-gold-conjugated anti-mouse antibody, which appears as black dots. Bar = 500 nm. Download FIG S4, PDF file, 14.7 MB. Copyright © 2018 Li et al. 2018 Li et al. This content is distributed under the terms of the Creative Commons Attribution 4.0 International license.

In addition to marked abnormalities in cell morphology, the *uut1*Δ mutant was highly susceptible to a range of stresses. It demonstrated temperature-sensitive growth (shown for solid and liquid media in Fig. 7B and Fig. S5, respectively), which was exacerbated by nutrient limitation (Fig. S5). Mutant cells also grew poorly in the presence of SDS or high salt (Fig. 7B). Heterologous expression of the human UDP-GlcA transporter, UGTrel7, did not restore growth under any of these conditions (Fig. S6).

10.1128/mBio.02319-17.5 FIG S5  Growth of the *uut1*Δ mutant (red) is restricted at 37°C and at 30°C under nutrient-limiting conditions (either the yeast medium YNB or mammalian tissue culture media DMEM and RPMI 1640) compared to wild type (blue) and the complemented mutant (green). Strains were grown overnight at 30°C in YPD medium, diluted to 10^5^ cells per ml in the media indicated, and incubated at 30 or 37°C with shaking. The results shown are the averages of three measurements. Download FIG S5, PDF file, 0.1 MB. Copyright © 2018 Li et al. 2018 Li et al. This content is distributed under the terms of the Creative Commons Attribution 4.0 International license.

10.1128/mBio.02319-17.6 FIG S6  The human UDP-GlcA transporter does not complement *uut1*Δ. *uut1*Δ and WT strains transformed with vector alone (vector) or vector expressing His-tagged UGTrel7 were grown overnight at 30°C in YPD medium with G418, and 5-μl volumes of serial dilutions (10-fold serial dilutions starting at 10^6^ cells per ml) were spotted and grown as indicated on medium containing G418. Images of the WT and *uut1*Δ strains were taken 3 and 6 days later as indicated in the figure. Download FIG S6, PDF file, 0.1 MB. Copyright © 2018 Li et al. 2018 Li et al. This content is distributed under the terms of the Creative Commons Attribution 4.0 International license.

Since pigment production correlates with resistance to environmental stress (44–46), we assayed the ability of the mutant strain to produce melanin on medium containing the precursor l-3,4-dihydroxyphenylalanine (l-DOPA). We observed no melanization, however, even after 7 days of growth (Fig. 7C). In all phenotypic studies, the complement restored growth or melanization to WT levels.

**FIG 8:**
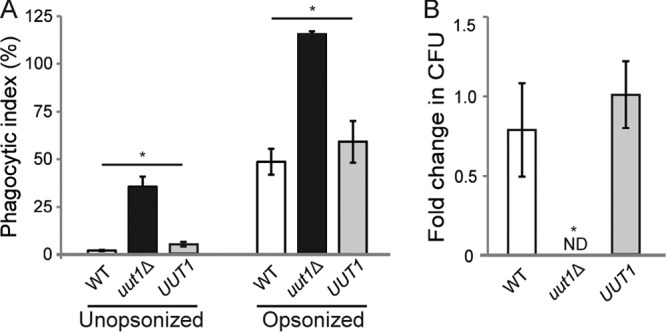
Cells lacking Uut1 are more efficiently phagocytosed and killed by human cells than by wild-type *C. neoformans*. (A) Phagocytic index (engulfed fungi/100 host cells) for fungi with or without serum opsonization. (B) Fold change in CFU (comparing results at 24 h to results at 1 h) after internalization of opsonized fungi by THP-1 cells. Data are means ± SEMs for three biological replicates. Values that are significantly different (*P* < 0.05) by one-way ANOVA and Tukey’s posthoc test are indicated by an asterisk. ND, not detected.

We next tested whether the observed mutant phenotypes would translate into aberrant interactions with host cells. Using an automated imaging method (47), we found that differentiated human monocytic cells (THP-1 cells) internalized the *uut1*Δ mutant at significantly higher rates than they internalized the WT, independent of serum opsonization (Fig. 8A). The mutant was also more susceptible to killing after internalization: host phagocytes completely cleared the *uut1*Δ mutant by 24 h, in contrast to stable levels of the WT and the complemented strain (*UUT1*; Fig. 8B).

**FIG 9:**
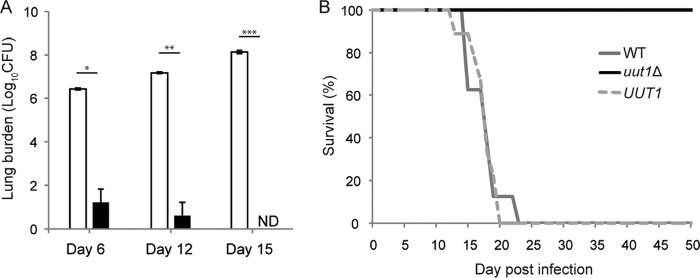
The *uut1*Δ mutant is severely attenuated for virulence. (A) Lung CFU of mice infected intranasally with 5 × 10^4^ cells of WT (white bars) or the *uut1*Δ mutant (black bars). Data shown are means ± SDs for three mice. Values that are significantly different by two-tailed Student’s *t* test are indicated by bars and asterisks as follows: *, *P* < 0.05; **, *P* < 0.01; ***, *P* < 0.001. ND, not detected. (B) Survival of mice infected as in panel A with the indicated strains (8 or 9 mice per strain).

Our data suggested that the *uut1*Δ mutant would poorly evade recognition and clearance by the host immune system and that it was unlikely to survive under host nutrient and temperature conditions. To test this *in vivo*, we inoculated mice with the WT, *uut1*Δ, or *UUT1* strain intranasally to mimic the natural route of infection. The *uut1*Δ mutant was cleared from the lungs by 15 days postinfection (dpi) (Fig. 9A), with no fungi detected in the brain or spleen at that time (Fig. S7). The WT or the complemented strain, in contrast, caused the mice to succumb to infection by 3 weeks postinoculation (Fig. 9B).

10.1128/mBio.02319-17.7 FIG S7  The *uut1*Δ mutant does not disseminate from the lung. The brain and spleen CFU at day 15 postinfection are shown. Values are means ± SDs for three mice. Download FIG S7, PDF file, 0.05 MB. Copyright © 2018 Li et al. 2018 Li et al. This content is distributed under the terms of the Creative Commons Attribution 4.0 International license.

## DISCUSSION

We have discovered the first fungal nucleotide sugar transporter that translocates UDP-GlcA. This protein, Uut1, is critical for *C. neoformans* virulence, likely due to its role in providing a key precursor for synthesis of the polysaccharide capsule and potentially other glycoconjugates, and it is also notable for its substrate specificity. Importantly, Uut1 has less than 12% identity at the protein level with its human counterpart, which also does not compensate for deficiencies in the fungal mutant (see Fig. S6 in the supplemental material).

Similar to other NSTs, Uut1 is localized to the secretory pathway. Our immunofluorescence studies suggest that this protein occurs mainly in the ER (Fig. 6), like the human transporter (UGTrel7) and in contrast to the more closely related, yet Golgi apparatus-localized, plant transporter (UUAT1) (33, 48). This is surprising, because we expect formation of mannose polymers to precede the addition of GlcA, and the mannose donor enters the secretory pathway in the Golgi apparatus (30, 49, 50). It may be that luminal UDP-GlcA progresses through the secretory pathway. Alternatively, the distribution of protein upon heterologous expression in *S. cerevisiae* may not accurately reflect the native localization, for example because of expression level or the lack of potential cryptococcal interaction partners. Furthermore, even if much of the protein is retrieved to the ER, perhaps to serve transport-independent functions, some fraction may remain in the Golgi apparatus (spatially separated from late Golgi proteins like Sec7) and carry out transport there. Further analysis to determine localization of the cryptococcal transporter must await the development of reliable subcellular imaging methods for *C. neoformans*.

Uut1 is unique in its specificity for UDP-GlcA among cryptococcal nucleotide sugars. Other known proteins that translocate UDP-GlcA also transport additional substrates *in vivo*: UGTrel7 and UUAT1 transport UDP-GalNAc and UDP-GalA, respectively (33, 48), while the *Drosophila* and *C. elegans* transporters, UST74c and SQV-7, recognize an even wider range of UDP-sugars (22, 51). Another notable feature of Uut1 is its affinity for UDP-GlcA, which is significantly above that of plant or human transporters, with a *K*_*m*_ of 0.6 μM compared to 1.5 mM for UUAT1 and 4 μM for UGTrel7 (33, 48). This low *K*_*m*_ relative to the measured cellular UDP-GlcA concentrations (Fig. 5B) suggests that Uut1 functions at a constant rate in both rich and nutrient-deficient media. Meeting increased demand for UDP-GlcA in the secretory pathway, for example under capsule-inducing conditions, thus requires more Uut1, which is achieved by upregulation at the transcriptional level (Fig. 6). The higher rates of UDP-GlcA transport out of the cytosol could then reduce UDP-Xyl production and consequent inhibition of Ugd1, balancing the system and maintaining stable UDP-GlcA levels (Fig. 5 and reported values above). In the absence of Uut1, there is likely an elevated pool of cytosolic UDP-GlcA due to the absence of transport and subsequent consumption, which leads to slightly increased UDP-Xyl production in that compartment and consequent feedback inhibition on Ugd1. Thus, total UDP-Glc, but not UDP-GlcA or UDP-Xyl, was significantly higher in *uut1*Δ cells than in WT cells.

Uut1 and the human transporter UGTrel7 overlap in subcellular localization, and both translocate UDP-GlcA, yet UGTrel7 does not rescue the *uut1*Δ mutant phenotype. At 550 amino acids, Uut1 is also roughly 70% longer than UGTrel7 and other NSTs, mainly due to a unique extended N-terminal cytosolic domain (Fig. S1) that is absent from the human and plant transporters and dispensable for transport activity (Fig. 4 and Fig. S2). It may be that this domain interacts with cryptococcal synthetic machinery to efficiently channel substrates into the luminal compartment or performs other functions specific to Uut1. Further studies of this unusual protein may elucidate functional differences that could be exploited for therapeutic intervention.

Cells lacking the donor of Xyl, an abundant capsule component, display capsule although the fibers are short and deformed (32, 40). Since GlcA is less abundant than Xyl in capsule polysaccharides, we originally expected to observe a similar phenotype in *uut1*Δ cells. We were therefore surprised to observe that these cells completely lacked capsule (Fig. 3 and Fig. S4). These unexpected results suggest that GlcA modification is a prerequisite for Xyl addition. This model is consistent with the greater variability in GXM and GXMGal of capsule Xyl residues compared to GlcA and their more distal position in GXMGal (11). An acapsular phenotype could also result if GlcA incorporation is required for the extension of GXM’s mannose backbone or to inhibit the degradation of unmodified mannose polymers.

Another possible explanation for the lack of capsule on *uut1*Δ cells is that GlcA modification is required for export of GXM and/or GXMGal, either directly by interacting with trafficking machinery or indirectly by enabling additional modifications (e.g., Xyl incorporation [76–78]) required for recognition. The mutant cells do contain unusual intracellular inclusions (Fig. 7 and Fig. S4), although their contents were not recognized by anti-GXM antibody (Fig. S4). (The possibility that aberrant polymers are made but are not recognized by the anti-GXM antibodies we tested remains, although none of these antibodies are reported to require GlcA for binding [52, 53].) Finally, GlcA modification could be required for function of a protein involved in capsule synthesis (see below).

We observed numerous defects in the growth and morphology of cells lacking Uut1, including cell wall disorganization and abnormal plasma membranes (Fig. 7 and Fig. S3). This suggests a role for GlcA modification beyond the capsule. Although GlcA has not been detected thus far in surveys of cryptococcal protein-linked glycans or glycolipids (54–57), the levels might be below the limits of detection of the methods used. Glucuronidation could also potentially occur in additional contexts (e.g., the cytosol). Further detailed characterization of cryptococcal glyconconjugates may elucidate such mechanisms.

The lack of a GlcA donor in the secretory pathway drastically influences cryptococcal interactions with the host. The resulting absence of the polysaccharide capsule may expose normally hidden immunogenic components (Fig. S3) (58), while aberrant glycosylation may also create novel immunoreactive epitopes. Both of these could lead to the increased recognition and internalization of the *uut1*Δ mutant by macrophages that we observe (Fig. 8). Internalized *uut1*Δ cells are also rapidly cleared both *in vitro* (Fig. 8) and *in vivo* (Fig. 9), likely facilitated by the reduced ability of the *uut1*Δ mutant to resist environmental stress. These observations suggest processes involving UDP-GlcA synthesis as a potential target for intervention, which might exploit the unique features of key proteins like Uut1 and the novel biology of the pathogen.

Our discovery of a highly specific, high-affinity fungal UDP-GlcA transporter has provided novel insights into cryptococcal biology. These studies have advanced our understanding of the localization and sequence of glycan biosynthetic events and supported the hypothesis that GlcA is incorporated into structures other than capsule and that it plays integral roles in maintaining cellular homeostasis. This work thus sets the stage for future studies in both cryptococcal pathogenesis and fundamental glycobiology.

## MATERIALS AND METHODS

### Sequence and phylogenetic analyses.

*uut1* was identified by BLASTP searches against *Cryptococcus neoformans* predicted proteins (Broad Institute; *Cryptococcus neoformans* var. *grubii* H99 database) using known UDP-GlcA transporters from *Arabidopsis thaliana* (accession no. NP_196036.1), *Caenorhabditis elegans* (accession no. NP_495436.1 and AT5G04160), *Homo sapiens* (accession no. NP_055954.1), and *Drosophila melanogaster* (accession no. NP_524126.1). Multiple-sequence alignment (MUSCLE [59]), phylogenetic analysis (PhyML [60]), and tree rendering (TreeDyn [61]) of Uut1, characterized UDP-GlcA transporters (listed above), and other known cryptococcal nucleotide sugar transporters (NSTs) was done using the online Phylogeny.fr program (http://www.phylogeny.fr/index.cgi) with default settings (62, 63). The putative protein topology of Uut1 was predicted using the Constrained Consensus TOPology prediction server (CCTOP; Institute of Enzymology, Budapest, Hungary) (64, 65) and visualized using Protter (http://wlab.ethz.ch/protter/start/) (66). The predicted endoplasmic reticulum (ER) localization signal was identified using LocSigDB (http://genome.unmc.edu/LocSigDB/) (67).

### Cell growth.

*C. neoformans* strains were grown at 30°C in YPD medium (1% [wt/vol] Bacto yeast extract, 2% [wt/vol] Bacto peptone, 2% [wt/vol] dextrose) with shaking (230 rpm) unless otherwise noted.

For phenotypic analysis, cells from cultures grown overnight (O/N; 16 to 18 h) were washed in sterile phosphate-buffered saline (PBS) and resuspended at 10^6^ cells/ml in PBS, and 5-μl aliquots of fivefold serial dilutions were plated and grown at 30°C or 37°C as indicated. Conditions tested included YPD plates containing 0.01% SDS, 1.2 M NaCl, 1.2 M KCl, Tris (pH 8.8), 1.5 M sorbitol, 0.05% Congo red (CR), or 2% calcofluor white (CFW). Samples were also tested on YNB medium (0.67% [wt/vol] yeast nitrogen base without amino acids, 2% [wt/vol] glucose, 2% [wt/vol] agar, 25 mM sodium succinate [pH 4.0]) supplemented with 0.5 mM hydrogen peroxide (H_2_O_2_) or sodium nitrite (NaNO_2_) to test oxidative and nitrosative stress sensitivity, respectively. Cell-associated melanin production was assayed by plating 5 μl of a solution of 10^6^ cells/ml on agar plates containing 8 mg/ml KH_2_PO_4_, 2 mg/ml glucose, 2 mg/ml l-glycine, 1 μg/ml d-biotin, 1 μg/ml thiamine, 0.92 mg/ml MgSO_4_ ⋅_ _7H_2_O, and 0.4 mg/ml l-3,4-dihydroxyphenylalanine (l-DOPA; Sigma-Aldrich).

To determine the growth rates of various strains, cells from O/N cultures were washed in sterile PBS and resuspended at 10^5^ cells/ml in 30 ml of YPD, YNB, Dulbecco modified Eagle medium (DMEM), or RPMI 1640 medium at 30°C or 37°C as indicated. Triplicate samples were taken at regular intervals and counted with a hemocytometer.

### *C. neoformans* strains and plasmids.

To generate the *uut1*Δ mutant, we replaced *UUT1* in the *C. neoformans* KN99α wild-type (WT) strain with a nourseothricin (NAT) resistance marker, using a split marker strategy (68). Transformants of interest were identified by resistance to NAT and validated by PCR verification of gene replacement. We used a similar strategy to replace the NAT deletion cassette with *UUT1* (amplified from KN99α cDNA) or *UGTREL7* (amplified from a pMKIT-neo hUGTrel7-HA plasmid [48]), in tandem with a Geneticin (G418) resistance marker. Transformants resistant to G418 and sensitive to NAT were verified by PCR.

### *Saccharomyces cerevisiae* localization.

*UUT1* was amplified from *C. neoformans* KN99α cDNA, cloned into the copper-inducible expression vector pYEScupFLAG*K* (26), and transformed into *S. cerevisiae* strain Sec7-3xGFP (from Benjamin S. Glick, University of Chicago) using lithium acetate. After O/N growth in synthetic complete (SC) medium minus uracil (URA), cultures were adjusted to 0.5 mM CuSO_4_ and cultured for 1 h. The cells were fixed in 1% paraformaldehyde for 30 min, washed, resuspended in a 0.1 M KPO_4_ (pH 6.5)–1.2 M sorbitol buffer, and then incubated for 15 min in buffer supplemented with β-mercaptoethanol (2% [vol/vol]) and zymolyase (100 μg/ml; Sigma-Aldrich). Fifteen-microliter aliquots of buffer-washed cells were then spotted onto polylysine-coated slides (Electron Microscopy Sciences), and the slides were incubated for 10 min and immediately plunged first in methanol for 5 min and then in acetone for 30 s. The samples were blocked with 5% goat serum in PBS for 30 min and stained O/N at 4°C with anti-FLAG (mouse antibody diluted 1:1,000; Invitrogen) and anti-Kar2p/BiP antibody (rabbit antibody 1:1,000; from Jeff Brodsky, University of Pittsburgh). Finally, cells were incubated for 2 h with Alexa Fluor 594-tagged goat anti-mouse IgG (Thermo Fisher Scientific), Alexa Fluor 488-tagged goat anti-rabbit IgG (Thermo Fisher Scientific), and 4′,6′-diamidino-2-phenylindole (DAPI) (Thermo Fisher Scientific), and viewed with a Zeiss Axioskop2 MOT Plus microscope (Carl Zeiss Microscopy, LLC). Where not specified, all steps were performed at room temperature (RT).

### Capsule induction and visualization.

Cultures of *C. neoformans* grown in YPD O/N were collected by centrifugation (3,000 × *g*, 5 min) and washed twice with sterile PBS. The cells were then resuspended in DMEM at 10^6^ cells/ml in T-75 tissue culture flasks or 24-well plates and incubated at 37°C with 5% CO_2_ for 24 h to induce production of capsule. Induced cells were collected, washed, and resuspended in PBS, mixed with 1.5 parts India ink (Chartpak, Inc.), and viewed with a Zeiss Axioskop2 MOT Plus microscope (Carl Zeiss Microscopy, LLC).

### GXM detection.

Cell wall-associated and shed GXM were visualized by fluorescence microscopy and immunoblotting, respectively. To visualize capsule on cells, the strains were induced as described above for 24 h, fixed for 1 h in 3.7% formaldehyde, washed in PBS, and then incubated for 1 h at RT with 1 mg/ml of anti-GXM monoclonal antibody (MAb) 3C2, 2H1, 3O2, 339, or F12D2 (from Thomas R. Kozel, University of Nevada School of Medicine) conjugated to Alexa Fluor 488. Stained cells were washed twice with PBS, resuspended in PBS, and examined on a Zeiss Axioskop 2 MOT Plus microscope. All samples from each experiment were imaged with identical acquisition settings. To analyze shed GXM, strains were induced for 90 min or 24 h before cells were removed by centrifugation. The supernatant fractions were then denatured with heat (60°C for 5 min), resolved on agarose gels, transferred onto a positively charged nylon membrane, and immunoblotted with 1 μg/ml anti-GXM MAb 3C2, 2H1, F12D2, or 339 as described in reference 69. The GXM content of the supernatant fractions was quantified by ELISA as described in reference 70 using MAb 339 and F12D2.

### Heterologous expression, reconstitution, and transport assays.

The *UUT1* coding region was synthesized into pUC57-Amp by Genewiz, amplified by PCR without the native stop codon, and introduced into the pENTR/SD/D-TOPO vector (Life Technologies) according to the manufacturer’s protocols to generate pENTR-*UUT1*. Recombination of the entry clone with destination vector pYES-DEST52 (Life Technologies) using LR clonase II (Life Technologies) produced a C-terminal His/V5 epitope fusion that was verified by sequencing before transformation into *S. cerevisiae* strain INVSc1 (Thermo Fisher Scientific). To verify heterologous protein expression, 2.5 µg of the proteoliposomes were resolved by SDS-PAGE and analyzed by immunoblotting with anti-V5 antibody (Thermo Fisher Scientific) as previously described (71). Reconstitution of microsomal proteins and transporter activity assays were also carried out as previously described (71). UDP-GlcA transport was measured at the UDP-GlcA concentrations and times indicated, and kinetic parameters were calculated by nonlinear regression using the Prism 6 application (GraphPad Software). Measured Uut1 content (see Table S2 in the supplemental material) was used to determine turnover rate.

10.1128/mBio.02319-17.9 TABLE S2  Uut1 content of proteoliposomes used for transport assays. Download TABLE S2, PDF file, 0.04 MB. Copyright © 2018 Li et al. 2018 Li et al. This content is distributed under the terms of the Creative Commons Attribution 4.0 International license.

### Quantification of nucleotide sugars by mass spectrometry.

Nucleotide sugars were extracted from approximately 50 mg of ground cells (wet weight) according to previous methods (72). Liquid chromatography-tandem mass spectrometry (LC-MS/MS) was performed using porous graphitic carbon as the stationary phase on a 1100 series high-performance liquid chromatography (HPLC) system (Agilent Technologies) and a 4000 QTRAP LC-MS/MS system (Sciex) equipped with a TurboIonSpray ion source using methods previously described (73). Four biological replicates were analyzed, each in duplicate.

### Fungal gene expression.

Wild-type cells cultured in YPD medium were induced for capsule as described above and sampled at 0, 1.5, 3, 8, and 24 h for RNA isolation and sequencing. See reference 74 for details.

### Electron microscopy.

Strains were grown in YPD medium or under capsule-inducing conditions, collected by centrifugation (3,000 × *g*, 5 min), fixed for 1 h at RT with 2% glutaraldehyde (Polysciences Inc.) in 100 mM phosphate buffer (pH 7.2), and then incubated for 1 h in 1% osmium tetraoxide (OsO_4_; Polysciences Inc.). Following dehydration with ethanol and propylene oxide, the cells were embedded in Eponate 12 resin (Tel Pella Inc.), and 70- to 90-nm sections were cut with a UCT ultramicrotome (Leica Microsystems Inc.). Sections were stained with uranyl acetate and lead citrate for visualization with a JEOL 1200EX transmission electron microscope (JEOL Inc.).

For immunoelectron microscopy, cells were fixed and labeled as described in reference 17. Briefly, induced cells were fixed in glutaraldehyde as described above, washed in citrate buffer (pH 6.0), and treated with lysing enzymes from *Trichoderma harzianum* (Sigma-Aldrich) in the same buffer for 30 min before being washed in 0.1 M phosphate buffer (pH 7.0), and postfixed in 1% OsO_4_. Ethanol-substituted samples were then substituted in propylene oxide and embedded in Eponate 12 resin. Sections were blocked with 5% fetal bovine serum (FBS) (Thermo Fisher Scientific) in piperazine-*N*,*N*′-bis(2-ethanesulfonic acid) (PIPES) buffer (pH 7.0) for 30 min, labeled with the anti-GXM MAb 3C2 for 1 h, washed in blocking buffer, and incubated with 12-nm-gold-conjugated goat anti-mouse IgG (Jackson Immuno Research). Sections were then washed in PIPES buffer and water, stained with uranyl acetate and lead citrate, and viewed with a JEOL JEM-1400Plus 120-kV transmission electron microscope (JEOL Inc.).

### Cell wall staining.

For eosin Y staining, O/N cultures were washed, diluted to 10^7^ cells/ml in McIlvaine’s buffer (pH 6.0), and incubated with 250 μg/ml eosin Y for 15 min. For the other dyes, the cells were washed, diluted to 10^7^ cells/ml in PBS, and stained for 15 min with 100 μg/ml calcofluor white (CFW) (fluorescent brightener 28; Sigma), 30 μg/ml concanavalin A (ConA)-fluorescein isothiocyanate (FITC) (Sigma), or 1:10,000 dilution Pontamine (Pontamine fast scarlet 4B; Bayer Co.). The cells were then washed in PBS and imaged with a Zeiss Axioskop2 MOT Plus microscope.

### Macrophage assays.

Macrophage phagocytosis and survival of fungal strains was quantified as described in reference 39. Briefly, cells were grown in YPD medium, collected by centrifugation, washed, and opsonized with human serum before incubation with differentiated THP-1 macrophages for 1 h. To measure fungal uptake by phagocytes, host cell cytosol and nuclei and fungal walls were stained, and samples were imaged on a Cytation3 plate reader (BioTek) and analyzed using IN Cell Developer Toolbox 1.9.2 (GE Healthcare Life Sciences). For survival assays, samples were washed twice with PBS and lysed either immediately or after a 24-h incubation, and the lysate was plated on YPD agar for CFU counts. Assay results for the *uut1*Δ mutant were compared to those for the wild-type and complemented strains by one-way analysis of variance (ANOVA) with Tukey’s posthoc test.

### Animal studies.

Fungal strains to be tested were cultured O/N in YPD medium, washed in sterile PBS, and diluted to 10^6^ cells/ml in sterile PBS. Four- to 6-week-old female A/JCr mice (National Cancer Institute) were then intranasally inoculated with 50-μl aliquots of each strain. Groups of three mice infected with the WT strain and three mice infected with the *uut1*Δ mutant were sacrificed at 6, 12, and 15 days postinoculation. Initial inocula and organ (lung, brain, spleen) homogenates were plated for CFU, and organ burden was analyzed by Student’s *t* test. Additional groups of eight mice were infected with WT, *uut1*Δ, and *UUT1* strains, weighed daily, and sacrificed once they lost >20% of their body weight relative to peak weight or at day 50. Survival curves were compared using a log rank test in GraphPad Prism. All studies were performed in compliance with institutional guidelines for animal experimentation.
