# Supplementary material for: UDP-Glucuronic Acid Transport Is Required for Virulence of Cryptococcus neoformans
Source: mBio. 2018 Jan 30;9(1):e02319-17. doi: 10.1128/mBio.02319-17 (PMC5790919; doi:10.1128/mBio.02319-17)
Supplement: FIG S1 [file mbo001183697sf1.pdf]

Supplemental Figure 1

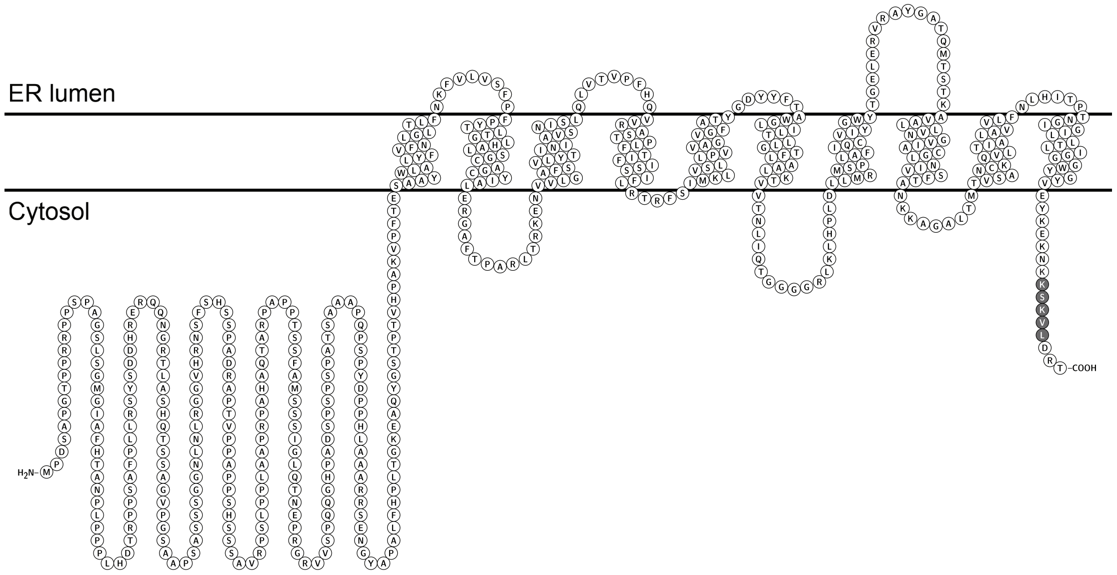

Fig. S1. Predicted secondary structure of Uut1 (550 amino acids), showing an extended N-terminal domain (amino acids 1-244), 10 predicted transmembrane domains, and a predicted C-terminal ER localization signal (KxKxx motif; dark grey).
