# Supplementary material for: UDP-Glucuronic Acid Transport Is Required for Virulence of Cryptococcus neoformans
Source: mBio. 2018 Jan 30;9(1):e02319-17. doi: 10.1128/mBio.02319-17 (PMC5790919; doi:10.1128/mBio.02319-17)
Supplement: TABLE S1 [file mbo001183697st1.pdf]

Table S1. Summary of GXM detection assays.

	ELISA (ng/mL, mean ± SEM)		GXM blotting				Capsule Staining
mAb / Strain	339	F12D2	2H1	3O2	339	F12D2	2H1
WT	31.9 ± 0.3	113.3 ± 12.9	+	+	+	+	+
*uut1*Δ	0 ± 0	0 ± 0	-	-	-	-	-
UUT1	25.2 ± 3.2	69.8 ± 7.7	+	+	+	+	+
*cap59*Δ	0 ± 0	0 ± 0	-	-	-	-	-
