# Supplementary material for: UDP-Glucuronic Acid Transport Is Required for Virulence of Cryptococcus neoformans
Source: mBio. 2018 Jan 30;9(1):e02319-17. doi: 10.1128/mBio.02319-17 (PMC5790919; doi:10.1128/mBio.02319-17)
Supplement: FIG S2 [file mbo001183697sf2.pdf]

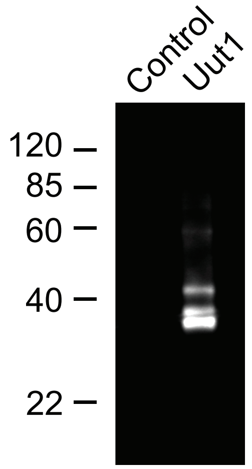

Fig. S2. Anti-V5 immunoblot of proteoliposomes prepared from *S. cerevisiae* expressing vector alone (control) or V5-tagged Uut1; 2.5 μg total protein per lane. Standards, in kDa, are shown at left. In *S. cerevisiae*, the expressed and active polypeptide is cleaved prior to amino acid 200.
