# Supplementary material for: UDP-Glucuronic Acid Transport Is Required for Virulence of Cryptococcus neoformans
Source: mBio. 2018 Jan 30;9(1):e02319-17. doi: 10.1128/mBio.02319-17 (PMC5790919; doi:10.1128/mBio.02319-17)
Supplement: FIG S3 [file mbo001183697sf3.pdf]

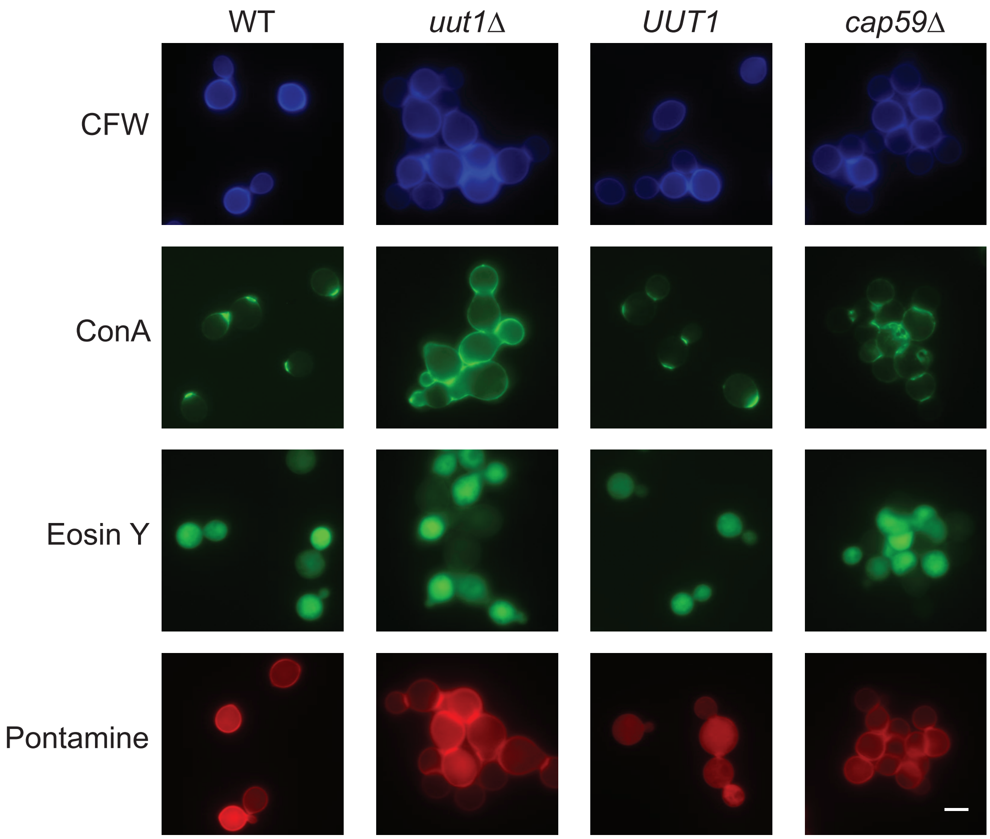

Fig. S3. Surface exposure of cell wall components. WT, *uut1*Δ, *UUT1*, and *cap59*Δ were grown and stained with CFW (binds chitin), Concanavalin A (binds mannoproteins), Eosin Y (binds chitosan), and Pontamine (binds unspecified cell wall components). Images are representative of two independent experiments. Scale bar = 5 μm.
