# Supplementary material for: UDP-Glucuronic Acid Transport Is Required for Virulence of Cryptococcus neoformans
Source: mBio. 2018 Jan 30;9(1):e02319-17. doi: 10.1128/mBio.02319-17 (PMC5790919; doi:10.1128/mBio.02319-17)
Supplement: FIG S4 [file mbo001183697sf4.pdf]

Supplemental Figure 4

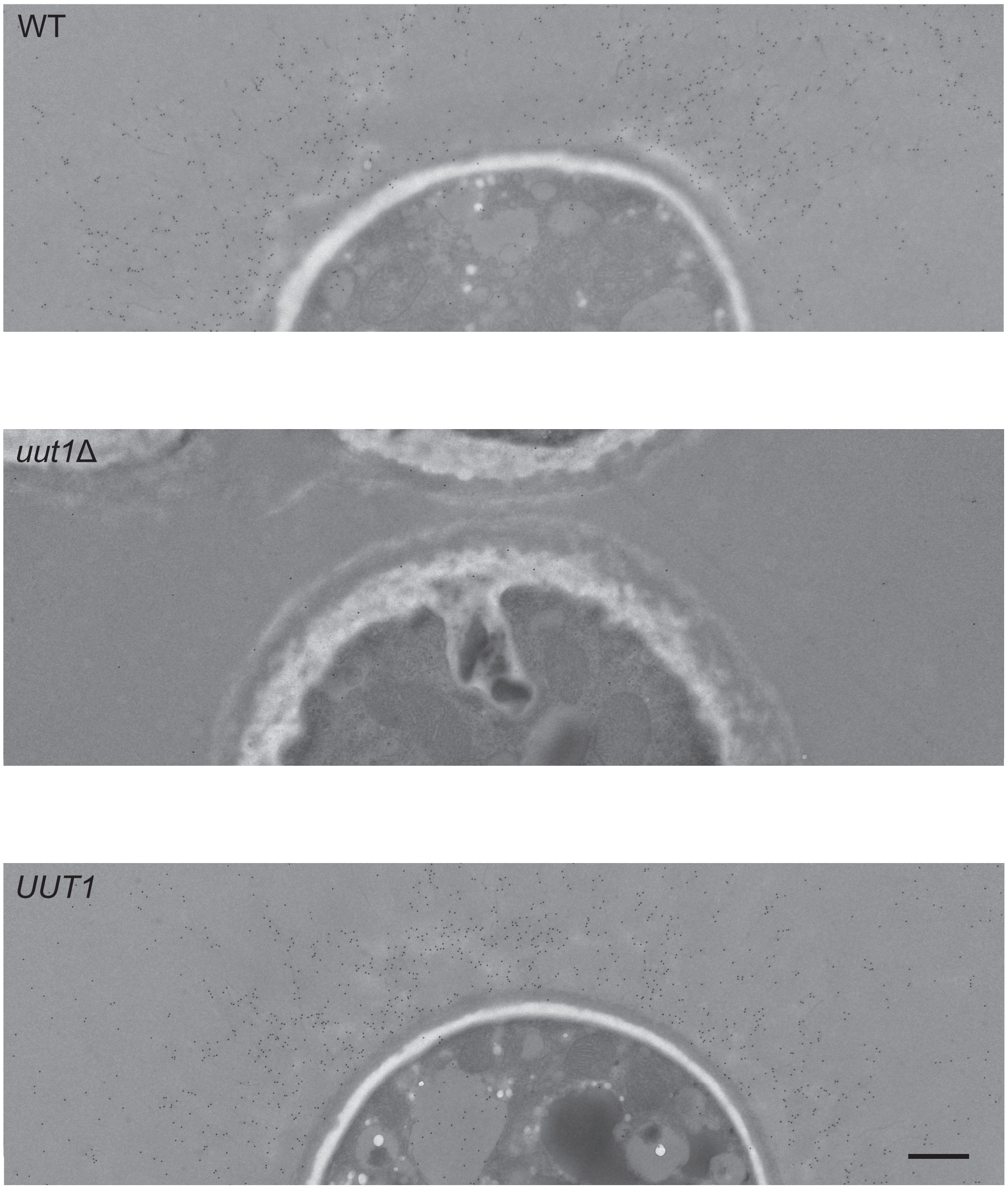

Fig. S4. GXM is not detectable within or around *uut1*Δ cells, in contrast to the abundant labeling of this capsule component on WT and complemented controls. Shown are electron micrographs of WT, *uut1*Δ, and *UUT1* strains grown for 24 h in nutrient-deficient media, which induces capsule production. Sections were labeled with anti-GXM mAb 3C2 and 12-nm gold-conjugated anti-mouse antibody, which appears as black dots (scale bar = 500 nm).
