# Supplementary material for: UDP-Glucuronic Acid Transport Is Required for Virulence of Cryptococcus neoformans
Source: mBio. 2018 Jan 30;9(1):e02319-17. doi: 10.1128/mBio.02319-17 (PMC5790919; doi:10.1128/mBio.02319-17)
Supplement: FIG S5 [file mbo001183697sf5.pdf]

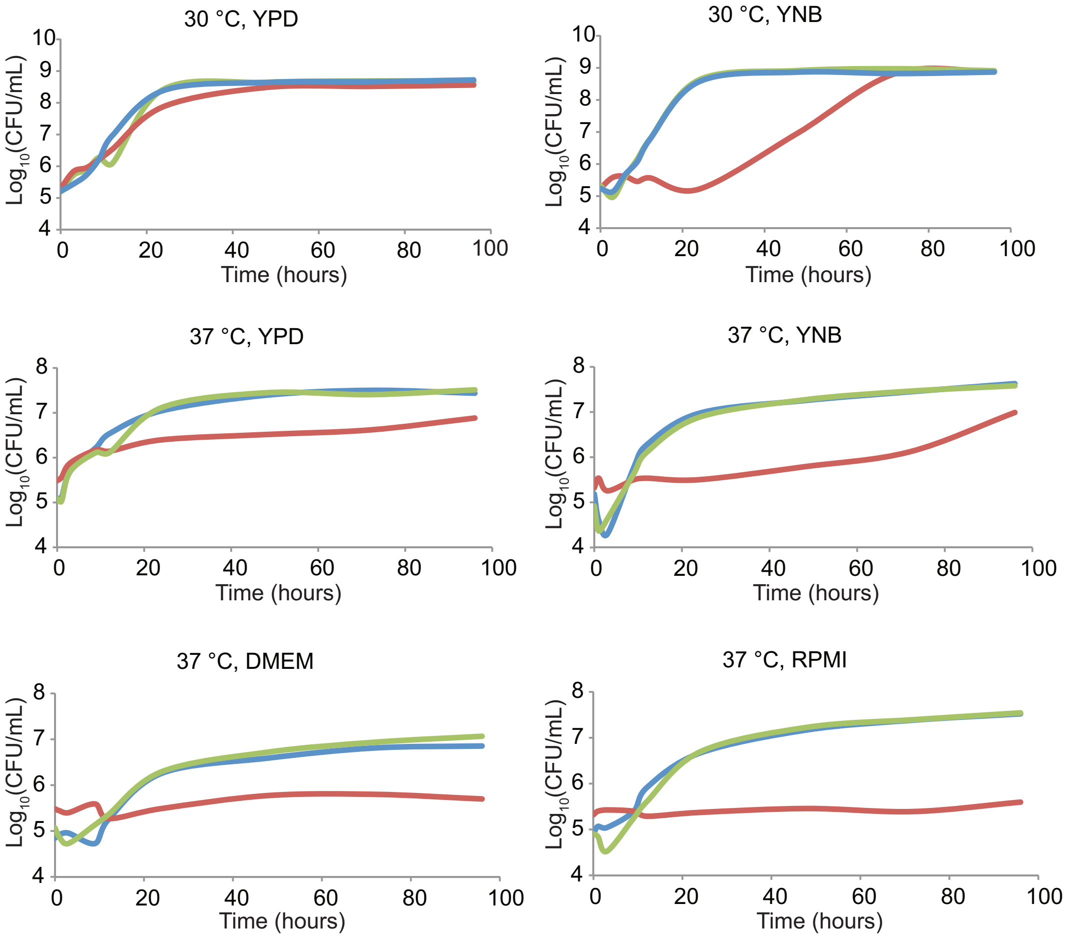

Fig. S5. Growth of *uut1*Δ (red) is restricted at 37 °C and at 30 °C under nutrient-limiting conditions (either the yeast medium YNB or mammalian tissue culture media DMEM and RPMI) compared to wild type (blue) and the complemented mutant (green). Strains were grown overnight at 30 °C in YPD, diluted to 10^5 cells per mL in the media indicated, and incubated at 30 or 37 °C with shaking. The results shown are the averages of three measurements.
