# Supplementary material for: UDP-Glucuronic Acid Transport Is Required for Virulence of Cryptococcus neoformans
Source: mBio. 2018 Jan 30;9(1):e02319-17. doi: 10.1128/mBio.02319-17 (PMC5790919; doi:10.1128/mBio.02319-17)
Supplement: FIG S6 [file mbo001183697sf6.pdf]

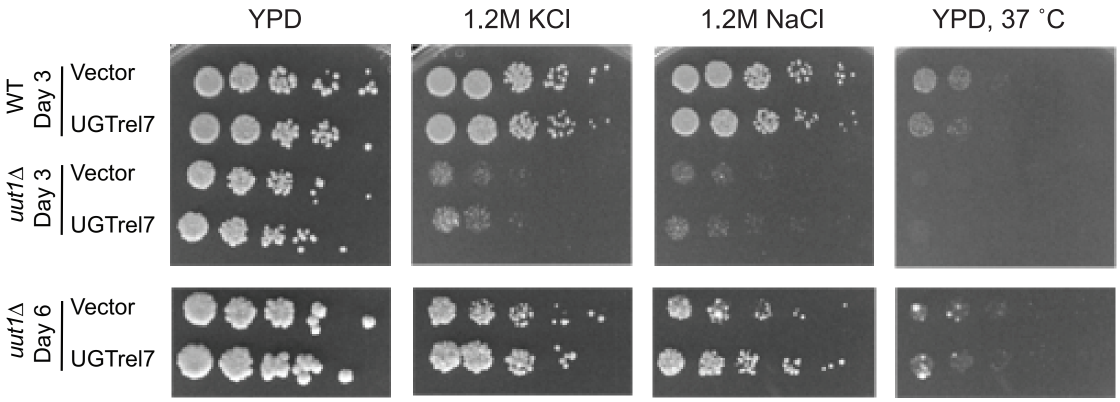

Fig. S6. The human UDP-GlcA transporter does not complement *uut1*Δ. *uut1*Δ and WT strains transformed with vector alone (vector) or vector expressing His-tagged UGTrel7 were grown overnight at 30°C in YPD with G418, and 5 μl of serial dilutions (10-fold starting at 10^6 cells per mL) were spotted and grown as indicated on medium containing G418. Images of the WT and *uut1*Δ strains were taken three and six days later as indicated.
