# Supplementary material for: UDP-Glucuronic Acid Transport Is Required for Virulence of Cryptococcus neoformans
Source: mBio. 2018 Jan 30;9(1):e02319-17. doi: 10.1128/mBio.02319-17 (PMC5790919; doi:10.1128/mBio.02319-17)
Supplement: FIG S7 [file mbo001183697sf7.pdf]

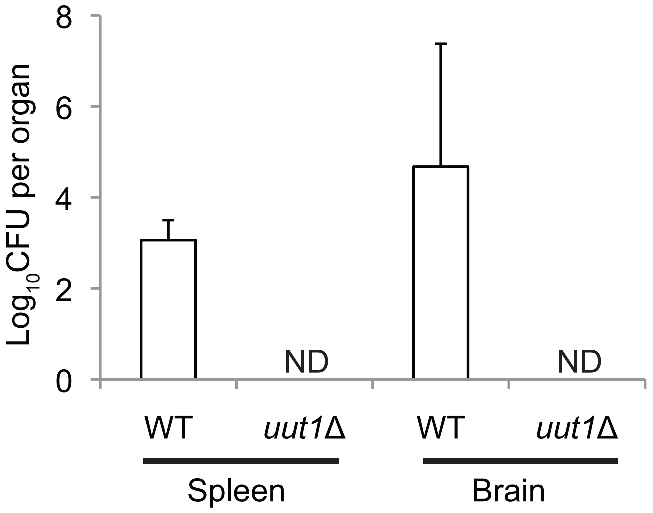

Fig. S7. *uut1*Δ does not disseminate from the lung. Brain and spleen CFU at day 15 post infection. Data shown is the mean ± SD for 3 mice.
