# Supplementary material for: UDP-Glucuronic Acid Transport Is Required for Virulence of Cryptococcus neoformans
Source: mBio. 2018 Jan 30;9(1):e02319-17. doi: 10.1128/mBio.02319-17 (PMC5790919; doi:10.1128/mBio.02319-17)
Supplement: TABLE S2 [file mbo001183697st2.pdf]

Table S2. Uut1 content of proteoliposomes used for transport assays.

	Molecular Mass (Da)	fmol*/5 μg	ng/5 μg	Total protein (%)
Uut1	63195.7	79.7 ± 4.1†	5.0 ± 0.3†	0.1 ± 0.0†

*Amount was estimated using LC-MS/MS (MRM) quantitation of a C-terminal peptide (SRGPFEGKPIPNPLLGLDSTR), and interpreted based on the molecular mass (including V5-tag and 6-His tags) estimated using the Compute pI/Mw tool at ExPASy (http://web.expasy.org/).

†Values represent the mean ± SD of n = 3.
